# The association between adult attained height and sitting height with mortality in the European Prospective Investigation into Cancer and Nutrition (EPIC)

**DOI:** 10.1371/journal.pone.0173117

**Published:** 2017-03-03

**Authors:** Norie Sawada, Petra A. Wark, Melissa A. Merritt, Shoichiro Tsugane, Heather A. Ward, Sabina Rinaldi, Elisabete Weiderpass, Laureen Dartois, Mathilde His, Marie-Christine Boutron-Ruault, Renée Turzanski-Fortner, Rudolf Kaaks, Kim Overvad, María-Luisa Redondo, Noemie Travier, Elena Molina-Portillo, Miren Dorronsoro, Lluis Cirera, Eva Ardanaz, Aurora Perez-Cornago, Antonia Trichopoulou, Pagona Lagiou, Elissavet Valanou, Giovanna Masala, Valeria Pala, Petra HM Peeters, Yvonne T. van der Schouw, Olle Melander, Jonas Manjer, Marisa da Silva, Guri Skeie, Anne Tjønneland, Anja Olsen, Marc J. Gunter, Elio Riboli, Amanda J. Cross

**Affiliations:** 1 School of Public Health, Imperial College London, London, United Kingdom; 2 Center for Public Health Sciences, National Cancer Center, Tokyo, Japan; 3 International Agency for Research on Cancer, Lyon, France; 4 Department of Community Medicine, Faculty of Health Sciences, University of Tromsø, The Arctic University of Norway, Tromsø, Norway; 5 Department of Research, Cancer Registry of Norway, Institute of Population-Based Cancer Research, Oslo, Norway; 6 Department of Medical Epidemiology and Biostatistics, Karolinska Institutet, Stockholm, Sweden; 7 Genetic Epidemiology Group, Folkhälsan Research Center, Helsinki, Finland; 8 Health Across Generations Team, CESP, Université Paris-Sud, UVSQ, INSERM, Université Paris-Saclay, Villejuif, France; 9 Division of Cancer Epidemiology; German Cancer Research Center (DKFZ), Heidelberg, Germany; 10 Aarhus University, Department of Public Health, Section for Epidemiology, Aarhus, Denmark; 11 Aalborg University Hospital, Department of Cardiology, Aalborg Hospital Science and Innovation Center, Aalborg, Denmark; 12 Public Health Directorate, Asturias, Spain; 13 Unit of Nutrition and Cancer, Cancer Epidemiology Research Program, Catalan Institute of Oncology, L'Hospitalet de Llobregat, Spain; 14 Escuela Andaluza de Salud Pública. Instituto de Investigación Biosanitaria ibs. Granada, Hospitales Universitarios de Granada/Universidad de Granada, Granada, Spain; 15 CIBER de Epidemiología y Salud Pública, Madrid, Spain; 16 Public Health Direction and Biodonostia-Ciberesp, Basque Regional Health Department, San Sebastian, Spain; 17 Unidad de Registro y Estadística de Mortalidad, Unit of Mortality Coding and Statistics, Servicio de Epidemiología, Consejería de Sanidad, Department of Epidemiology, Murcia’s Regional Health Council, Murcia, Spain; 18 Navara Public Health Institute, Pamplona, Spain; 19 IdiSNA, Navara Institute for Health Research, Pamplona, Spain; 20 Cancer Epidemiology Unit, Nuffield Department of Population Health, University of Oxford, Oxford, United Kingdom; 21 Hellenic Health Foundation, Athens, Greece; 22 WHO Collaborating Center for Nutrition and Health, Unit of Nutritional Epidemiology and Nutrition in Public Health, Department of Hygiene, Epidemiology and Medical Statistics, University of Athens Medical School, Athens, Greece; 23 Department of Epidemiology, Harvard School of Public Health, Boston, United States of America; 24 Molecular and Nutritional Epidemiology Unit, Cancer Research and Prevention Institute–ISPO, Florence, Italy; 25 Epidemiology and Prevention Unit, Fondazione IRCCS Istituto Nazionale dei Tumori, Milan, Italy; 26 Julius Center for Health Sciences and Primary Care, University Medical Center Utrecht, Utrecht, The Netherlands; 27 Department of Clinical Sciences, Lund University, Malmö, Sweden; 28 Department of Surgery, Skane University Hospital Malmo Lund University, Malmö, Sweden; 29 Danish Cancer Society Research Center, Copenhagen, Denmark; Medizinische Universitat Innsbruck, AUSTRIA

## Abstract

Adult height and sitting height may reflect genetic and environmental factors, including early life nutrition, physical and social environments. Previous studies have reported divergent associations for height and chronic disease mortality, with positive associations observed for cancer mortality but inverse associations for circulatory disease mortality. Sitting height might be more strongly associated with insulin resistance; however, data on sitting height and mortality is sparse. Using the European Prospective Investigation into Cancer and Nutrition study, a prospective cohort of 409,748 individuals, we examined adult height and sitting height in relation to all-cause and cause-specific mortality. Height was measured in the majority of participants; sitting height was measured in ~253,000 participants. During an average of 12.5 years of follow-up, 29,810 deaths (11,931 from cancer and 7,346 from circulatory disease) were identified. Hazard ratios (HR) with 95% confidence intervals (CI) for death were calculated using multivariable Cox regression within quintiles of height. Height was positively associated with cancer mortality (men: HR_Q5 vs. Q1_ = 1.11, 95%CI = 1.00–1.24; women: HR_Q5 vs. Q1_ = 1.17, 95%CI = 1.07–1.28). In contrast, height was inversely associated with circulatory disease mortality (men: HR_Q5 vs. Q1_ = 0.63, 95%CI = 0.56–0.71; women: HR_Q5 vs. Q1_ = 0.81, 95%CI = 0.70–0.93). Although sitting height was not associated with cancer mortality, it was inversely associated with circulatory disease (men: HR_Q5 vs. Q1_ = 0.64, 95%CI = 0.55–0.75; women: HR_Q5 vs. Q1_ = 0.60, 95%CI = 0.49–0.74) and respiratory disease mortality (men: HR_Q5 vs. Q1_ = 0.45, 95%CI = 0.28–0.71; women: HR_Q5 vs. Q1_ = 0.60, 95%CI = 0.40–0.89). We observed opposing effects of height on cancer and circulatory disease mortality. Sitting height was inversely associated with circulatory disease and respiratory disease mortality.

## Introduction

Poor nutrition, illness and early life exposures may contribute to ill health in later life [1–3]; however, there is a paucity of data to explore such associations in prospective cohorts with extended follow-up of children. Adult height is an easily measured variable, and is thought to reflect both genetic and environmental factors including nutrition, physical and social environments in early life [4, 5].

The association between height and mortality has been investigated in previous studies. A meta-analysis of 121 cohort studies comprising over 1 million participants reported that height was inversely associated with risk of death from circulatory diseases such as coronary disease, stroke and heart failure [6]. In contrast, height was positively associated with risk of death from melanoma and cancers of the pancreas, endocrine and nervous systems, ovary, breast, prostate, colorectum, blood and lung [6]. Despite many studies investigating overall height and mortality, there have been few studies examining the association between sitting height and mortality. Higher sitting height is of interest because compared with adult height, sitting height may be more strongly positively associated with insulin resistance [7], and is positively associated with lung function, independently of height [8]; therefore, the effects of sitting height on mortality might be different from those of overall height. One cohort study reported that sitting height was positively associated with cancer mortality and inversely associated with death from circulatory disease [9], but others showed no association [7, 10–12].

To further knowledge on the association of height and health outcomes among adults, we examined whether adult height and sitting height were associated with overall and cause-specific mortality in a large prospective cohort of approximately half a million men and women from 10 European countries.

## Methods

### Study cohort

The European Prospective Investigation into Cancer and Nutrition (EPIC) study includes 23 centres within 10 European countries (Denmark, France, Germany, Greece, Italy, the Netherlands, Norway, Spain, Sweden, and the United Kingdom (UK)). Most centres recruited from the general population living in defined towns and provinces. In Florence (Italy) and Utrecht (the Netherlands), however, participants undergoing breast cancer screening were recruited; parts of the Italian and Spanish cohorts were recruited among blood donors and their spouses; most of the Oxford cohort (UK) consisted of vegetarian and health-conscious volunteers; and female members of the health insurance scheme for state school employees were recruited in France. Between 1992 and 2000, 521,457 individuals (approximately 70% women, mostly 20–70 years old) were enrolled after providing written informed consent. Ethical approval for the EPIC study was obtained from the review boards of the International Agency for Research on Cancer (IARC) and local participating centres. The cohort characteristics have been described in detail elsewhere [13, 14].

### Exposure assessment

At recruitment, standardized questionnaires on lifestyle, demographic information and personal history were collected [13, 14]. Height was measured in participating centres to the nearest 0.1, 0.5, or 1.0 cm in participants without shoes on [15]. Norway was the only centre in which height was all self-reported; furthermore, height was only measured in 29% of the French and 13% of the Oxford cohort with the remainder of the participants self-reporting their height. Self-reported data on height tends to be overestimated, with the degree of overestimation being larger for shorter individuals and this also depends on age [15]. The self-reported data from the Oxford cohort were adjusted using earlier described sex-specific regression equations that incorporated age [15]; this was not done for the French cohort because the interval between the self-reported data and measurement was considered to be too long to do so reliably, thus only those participants with measured data were included. Sitting height was measured (the minimum unit was 0.1cm) in over 90% of participants in six countries (Italy, Spain, the Netherlands, Germany, Greece, Denmark) and in 29% of French participants.

### Follow-up and endpoint assessment

Vital status, causes and dates of death were ascertained from population registries in Denmark, Italy (except Naples), the Netherlands, Norway, Spain, Sweden, and the UK. In Germany, Greece and Naples, this information was obtained by follow-up mailings or inquiries to municipal registries, regional health departments, physicians, and hospitals, and also by directly contacting their next-of-kin. In France, the causes and dates of death were obtained from the French Epidemiological Center for the Medical Causes of Death (CépiDc, Inserm).

Mortality data were coded according to the 10th revision of the International Classification of Diseases (ICD-10). All-cause mortality included deaths from external causes. The codes for the underlying cause of death were classified as follows: circulatory (ICD-10: I00-I99), cancer (C00-C97), respiratory (J00-J99), other or not reported (all other codes). Additionally, cancers were classified as smoking-related cancers [16] (oral cavity (C00-06), pharynx (C10), nasopharynx (C11-13), oesophagus (C15), stomach (C16), colorectal (C18-20), liver (C22), bile duct (C24), pancreas (C25), larynx and lung (C32-34), uterine cervix (C53), ovarian (C56), kidney and renal pelvis, ureter and bladder (C64-68), and myeloid leukaemia (C92) [16]) and non-smoking-related cancers (all other cancers). Furthermore, circulatory disease was subdivided into ischaemic heart disease (I20-I25), myocardial infarction (I21), cerebrovascular disease (I60-I69), haemorrhagic stroke (I60-I62) and ischemic stroke (I63).

### Statistical analysis

From the 521,457 participants recruited, those in a subsample in France (n = 52,809) and in the whole cohort in Norway (n = 37,185) were excluded because measured height was unavailable. Additionally, participants with missing questionnaire data (n = 1,286), missing dietary data (n = 6,627), missing all potential confounders (n = 3,127), without dates of death or follow-up information (n = 542) and those within the lowest and highest 1% of the cohort distribution of the ratio of reported total energy intake to energy requirements (n = 10,133) were excluded [17]. The final analytic cohort consisted of 409,748 individuals. For the analysis of sitting height, participants whose sitting height was not assessed were also excluded, leaving an analytic cohort for sitting height of 253,427 individuals.

Cox proportional hazard regression models with age as the underlying time scale were used to estimate hazard ratios (HR) and 95% confidence intervals (CI) for the association between height, sitting height and mortality risk. Height and sitting height were analysed as categorical variables defined by quintiles, and rounded off to the nearest 1 cm, and as continuous variables. Time at risk was estimated from the date of recruitment to the date of death, emigration, loss to follow-up, or the end of follow-up (a maximum through 2010, depending on centre), whichever occurred first. To control for differences in questionnaire design and follow-up procedures, all models were stratified by study centre. Models were further stratified by age at recruitment (continuous) to allow the form of the baseline hazard functions to vary across ages. All models were fitted for men and women separately, and adjusted for weight (kg, quintiles), combined recreational and household physical activity (inactive, moderately inactive, moderately active, active, unknown), alcohol consumption (0, >0–4, 5–14, 15–29, 30–59, ≥ 60 g/day), smoking status (never, former smokers (who quit <10 or ≥10 years ago), current smokers (1–14, 15–24 or ≥25 cigarettes per day), current smoker but amount missing (unknown smoking status), education level (none/primary school, technical or professional school, secondary school, university degree, not specified/missing) and energy intake (kcals/day, continuous). Moreover, the Cox models for women were further adjusted for menopausal status (pre, post-, peri-menopausal or unknown) and menopausal hormone use (yes, no, unknown). Potential non-linearity of the dose-response relationship was investigated using restricted cubic spline regression with knots placed at the 5^th^, 25^th^, 75^th^ and 95^th^ percentiles of height and corresponding likelihood ratio tests to compare the goodness-of-fit of the models with and without the spline terms [18, 19]. Because linearity could indeed be assumed, we computed a test for trend based on models with height and sitting height as a continuous variable.

Finally, we conducted interaction analyses to examine the relation between sitting height and overall height in relation to mortality; for these analyses we categorized height and sitting height into tertiles (low, middle and high) and examined risks within each combined strata and calculated a P-value for the interaction term. In addition, we examined interaction terms for height (as a continuous variable, per 5cm increment) and all-cause and cause-specific mortality according to country, age at recruitment, body mass index (BMI), smoking status and alcohol intake.

All reported P-values were two-sided and were regarded as statistically significant if P<0.05. The potential for multiple comparisons was addressed by examining Bonferroni correction; p = 0.004 (0.05/13 variables). All analyses were performed using Statistical Analysis Software (SAS version 9.1, SAS Institute, Cary, NC).

## Results

After an average follow-up of 12.5 years (range 0.01–17.8), 29,810 participants (15,320 men and 14,490 women) had died from any cause among all 409,748 participants. Out of all deaths with a reported cause (n = 25,526), major causes were cancer (n = 11,931), diseases of the circulatory system (n = 7,346) and the respiratory tract (n = 1,266). Among participants with data on sitting height, there were 15,630 all-cause deaths, 6,909 deaths from cancer, and 3,656 deaths from circulatory diseases. Participants who were taller, compared with those who were shorter, were younger, heavier, had higher energy intakes and were more physically active. The proportion of current smokers was lower in taller men but there was a higher proportion of current smokers in taller women; furthermore, taller women were more likely to have a higher education level, consume higher levels of alcohol, to be premenopausal, and among post-menopausal women, were more likely to use menopausal hormones (Table 1). After excluding individuals without data for sitting height, the characteristics were similar to those with measured height (data not shown).

**Table 1:** Baseline characteristics according to height.

Characteristic	Height									
	<168cm (n = 24,624)		168-172cm (n = 32,317)		173-175cm (n = 23,126)		176-180cm (n = 35,141)		≧181cm (n = 29,754)	
	%	Median (10–90%)	%	Median (10–90%)	%	Median (10–90%)	%	Median (10–90%)	%	Median (10–90%)
MEN										
Age at recruitment (years)		55.8 (42.9–67.5)		54.0 (41.1–64.9)		53.3 (40.1–64.2)		52.3 (39.1–63.3)		50.7 (32.7–61.6)
Weight (g)		73.0 (61.4–87.0)		77.0 (65.5–91.2)		79.4 (67.9–94.3)		81.8 (70.0–97.2)		86.5 (73.6–103.5)
Education										
None	13		5		2		1		0.3	
Primary school completed	43		35		31		25		18	
Technical/professional school completed	18		23		25		27		26	
Secondary school completed	9		12		13		14		17	
University	14		22		26		31		37	
Missing	3		3		3		3		2	
Smoking status										
Never smokers	30		31		32		34		37	
Former smokers	37		38		38		37		34	
Time since stopped smoking (years)		13.0 (2.5–31.0)		14.5 (3.0–31.0)		14.5 (2.5–31.0)		15.0 (2.5–30.5)		14.0 (2.5–29.0)
Duration of smoking (years)		23 (8–40)		21 (7–38)		20 (6–37)		20 (6–36)		18 (5–34)
Current smokers	32		30		30		28		28	
No. of cifarettes/day		18 (4–31)		18 (4–30)		17 (5–30)		17 (4–30)		15 (4–30)
Duration of smoking (years)		34.5 (22.5–48)		34.5 (21–46)		34 (20.5–45.5)		33.5 (19.5–45)		32 (14.5–43.5)
Missing	2		2		1		1		1	
Alcohol consumption (g/day)		12.3 (0–5.19)		12.6 (0.4–51.0)		12.6 (0.6–50.9)		12.5 (0.8–48.6)		12.3 (0.9–48.1)
Non-consumers	11		7		6		5		4	
Physical activity										
Active	21		24		25		25		26	
Total energy intake (kcal/day)		2,262 (1,543–3,201)		2,315 (1,599–3,248)		2,337 (1,612–3,262)		2,351 (1,627–3,298)		2,439 (1,687–3,394)
	<156cm (n = 48,547)		156-159cm (n = 51,049)		160-162cm (n = 47,088)		163-167cm (n = 66,397)		≧168cm (n = 51,705)	
	%	Median (10–90%)	%	Median (10–90%)	%	Median (10–90%)	%	Median (10–90%)	%	Median (10–90%)
WOMEN										
Age at recruitment (years)		54.6 (40.8–66.8)		52.7 (38.6–64.6)		51.7 (37.5–63.8)		51.3 (36.4–63.1)		50.0 (30.4–60.9)
Weight (g)		61.7 (50.1–78.1)		62.5 (51.6–79.8)		63.8 (53.1–81.0)		65.5 (55.0–82.8)		68.7 (58.2–86.0)
Education										
None	18		7		3		2		0.4	
Primary school completed	40		32		27		22		15	
Technical/professional school completed	13		21		25		28		30	
Secondary school completed	13		16		18		19		21	
University	11		18		22		24		31	
Missing	4		6		5		5		3.6	
Smoking status										
Never smokers	67		59		55		52		50	
Former smokers	16		21		24		25		27	
Time since stopped smoking (years)		14.0 (2.5–31.0)		14.0 (2.5–30.0)		14.0 (2.5–29.5)		14.0 (2.5–29.0)		13.0 (2.0–27.5)
Duration of smoking (years)		17.0 (4.0–34.0)		16.0 (4.0–33.0)		16.0 (4.0–32.0)		15.0 (4.0–32.0)		14.0 (4.0–31.0)
Current smokers	16		19		20		21		23	
No. of cifarettes/day		11.0 (3.0–21.0)		11.0 (3.0–21.0)		11.0 (3.0–21.0)		12.0 (3.0–21.0)		12.0 (3.0–21.0)
Duration of smoking (years)		27.0 (10.5–42.0)		28.5 (15.5–42.5)		29.5 (16.0–42.5)		30.0 (15.5–42.0)		29.0 (12.5–40.5)
Missing	1		1		1		1		1	
Alcohol consumption (g/day)		1.3 (0–17.7)		2.8 (0–20.7)		3.8 (0–22.7)		4.5 (0–23.8)		5.6 (0.2–25.6)
Non-consumers	20		19		14		11		8	
Physical activity										
Active	9		14		17		19		23	
Total energy intake (kcal/day)		1,808 (1,231–2,586)		1,852 (1,282–2,618)		1,873 (1,303–2,637)		1,886 (1,317–2,642)		1,923 (1,349–2,678)
Menopausal status										
Premenopausal	28		33		35		36		44	
Postmenopausal	55		49		46		44		37	
Perimenopausal	12		14		15		17		17	
Surgical postmenopausal	5		4		4		3		2	
Use of menopausal hormone										
yes	18		22		24		25		23	
Missing	5		8		9		11		11	

Tables 2 and 3 shows the HRs for height and all-cause and cause-specific mortality in men and women, respectively. There was a statistically significant linear inverse association between height and all-cause mortality in men (HR_Q5 vs. Q1_ = 0.85, 95%CI = 0.80–0.91, p for trend<0.01), but no association was observed in women (HR_Q5 vs. Q1_ = 1.01, 95%CI = 0.95–1.08, p for trend = 0.66). We observed a positive association between height and death from cancer in both sexes (HR_Q5 vs. Q1_ = 1.11, 95%CI = 1.00–1.24, p for trend = 0.08 in men; HR_Q5 vs. Q1_ = 1.17, 95%CI = 1.07–1.28, p for trend<0.01 in women). HRs for smoking-related cancers and non-smoking-related cancers were not substantially different (Table 3). In contrast, height was inversely associated with the risk of death from circulatory disease in both sexes (HR_Q5 vs. Q1_ = 0.63, 95%CI = 0.56–0.71, p for trend<0.01 in men; HR_Q5 vs. Q1_ = 0.81, 95%CI = 0.70–0.93, p for trend<0.01 in women). Furthermore, height was inversely associated with ischaemic heart disease and myocardial infarction in both men and women, as well as cerebrovascular disease mortality in men only. There was no association between height and death from stroke or respiratory diseases in men or women. Excluding subjects with a past history of cancer, cardiovascular disease or diabetes (n = 38,760) yielded similar results (data not shown).

**Table 2:** Hazard ratios* and 95% confidence intervals for all cause and cause-specific mortality according to height in men.

			Height						
Cause of death			<168 cm	168-<173 cm	173-<176 cm	176-<181 cm	≧181cm	P for linear trend†	HR (95% CI) per 5 cm increase in height
All-cause mortality		Person-years	293,986	390,290	280,389	431,003	369,328		
		Deaths	3,416	3,753	2,464	3,311	2,376		
		HR (95% CI)	1	**0.93 (0.89–0.98)**	**0.91 (0.86–0.96)**	**0.86 (0.81–0.91)**	**0.85 (0.80–0.91)**	**<0.01**	**0.96 (0.94–0.97)**
Cause-specific mortality		Person-years	277,037	363,393	260,139	399,967	344,047		
Cancer		Deaths	1,112	1,338	905	1,249	916		
		HR (95% CI)	1	1.05 (0.97–1.15)	1.07 (0.97–1.17)	1.06 (0.96–1.17)	**1.11 (1.00–1.24)**	0.08	**1.03 (1.00–1.05)**
	Smoking-related cancer	Deaths	453	574	373	519	389		
		HR (95% CI)	1	1.26 (1.05–1.52)	1.21 (0.98–1.50)	1.30 (1.06–1.59)	1.04 (0.83–1.31)	0.93	0.99 (0.95–1.04)
	Non smoking-related cancer	Deaths	659	764	532	730	527		
		HR (95% CI)	1	0.95 (0.82–1.09)	0.94 (0.80–1.10)	1.07 (0.91–1.25)	0.86 (0.72–1.03)	0.36	0.98 (0.95–1.02)
Circulatory disease		Deaths	1,166	1,065	715	845	608		
		HR (95% CI)	1	**0.79 (0.73–0.87)**	**0.78 (0.71–0.87)**	**0.64 (0.58–0.72)**	**0.63 (0.56–0.71)**	**<0.01**	**0.88 (0.86–0.90)**
	Ischaemic heart disease	Deaths	632	594	412	483	323		
		HR (95% CI)	1	**0.75 (0.66–0.84)**	**0.75 (0.65–0.86)**	**0.59 (0.52–0.68)**	**0.54 (0.46–0.63)**	**<0.01**	**0.86 (0.83–0.89)**
	Myocardial infarction	Deaths	319	311	207	267	172		
		HR (95% CI)	1	**0.78 (0.66–0.92)**	**0.75 (0.62–0.91)**	**0.64 (0.53–0.77)**	**0.54 (0.43–0.67)**	**<0.01**	**0.86 (0.82–0.90)**
	Cerebrovascular disease	Deaths	238	182	112	124	95		
		HR (95% CI)	1	0.81 (0.66–1.00)	0.80 (0.62–1.03)	**0.65 (0.50–0.84)**	**0.73 (0.54–0.99)**	**<0.01**	**0.91 (0.86–0.97)**
	Haemorrhagic stroke	Deaths	61	59	34	45	44		
		HR (95% CI)	1	0.80 (0.54–1.18)	0.65 (0.41–1.04)	0.61 (0.38–0.96)	0.73 (0.44–1.20)	0.13	**0.89 (0.79–0.99)**
	Ischemic stroke	Deaths	33	21	12	26	17		
		HR (95% CI)	1	0.47 (0.26–0.85)	0.36 (0.18–0.74)	0.60 (0.33–1.11)	0.55 (0.27–1.14)	0.20	0.85 (0.72–1.01)
Respiratory disease		Deaths	191	165	98	118	66		
		HR (95% CI)	1	0.96 (0.77–1.21)	1.01 (0.77–1.32)	0.95 (0.72–1.24)	0.87 (0.62–1.23)	0.49	0.97 (0.90–1.04)
Other cause of death		Deaths	595	624	389	567	431		
		HR (95% CI)	1	**0.86 (0.76–0.97)**	**0.77 (0.67–0.88)**	**0.76 (0.67–0.88)**	**0.74 (0.64–0.87)**	**<0.01**	**0.92 (0.89–0.95)**

* Hazard ratios (HR) and 95% confidence intervals (95% CI) estimated in a Cox regression model stratified by centre, 1-year age and 5-year birth cohort categories, adjusted for education level (none,/primary school completed, technical/professional school, secondary school, university degree, not specified), smoking status (never smoker, former smoker who quit <10 years ago, former smoker who quit >10 years ago, former smoker, unknown when quit, current smoker of 1–14 cigarettes a day, current smoker of 15–24 cigarettes a day, current smoker of > = 25 cigarettes a day, current smoker but amount missing, smoking status unknown), physical activity (inactive, moderately inactive, moderately active, active), alcohol consumption (0, 0-<5, 5-<15, 15-<30, 30-<60, > = 60 g/day), weight (quintiles), intake of energy (kcals/day, continuous).

† Median values of each category as continuous variable (cm).

**Table 3:** Hazard ratios* and 95% confidence intervals for all cause and cause-specific mortality according to height in women.

			Height						
Cause of death			<156 cm	156-<160 cm	160-<163 cm	163-<168 cm	≧168cm	P for linear trend†	HR (95% CI) per 5 cm increase in height
All-cause mortality		Person-years	590,468	628,657	581,655	680,731	791,060		
		Deaths	3,167	2,884	2,695	2,879	2,865		
		HR (95% CI)	1	0.97 (0.92–1.02)	1.04 (0.99–1.10)	0.98 (0.93–1.04)	1.01 (0.95–1.08)	0.66	1.00 (0.99–1.02)
Cause-specific mortality		Person-years	554,304	587,926	543,440	635,688	745,828		
Cancer		Deaths	1,138	1,254	1,201	1,335	1,483		
		HR (95% CI)	1	1.07 (0.98–1.16)	1.14 (1.04–1.24)	1.09 (0.99–1.19)	**1.17 (1.07–1.28)**	**<0.01**	**1.05 (1.02–1.07)**
	Smoking-related cancer	Deaths	503	512	542	540	551		
		HR (95% CI)	1	0.97 (0.81–1.17)	1.08 (0.89–1.31)	0.98 (0.81–1.19)	0.98 (0.80–1.20)	0.79	1.00 (0.95–1.04)
	Non smoking-related cancer	Deaths	635	742	659	795	932		
		HR (95% CI)	1	1.12 (0.96–1.30)	1.11 (0.95–1.30)	1.00 (0.86–1.17)	1.10 (0.94–1.28)	0.62	1.01 (0.97–1.05)
Circulatory disease		Deaths	842	638	506	521	440		
		HR (95% CI)	1	0.95 (0.85–1.06)	0.92 (0.82–1.04)	**0.86 (0.76–0.98)**	**0.81 (0.70–0.93)**	**<0.01**	**0.94 (0.91–0.97)**
	Ischaemic heart disease	Deaths	303	233	179	188	135		
		HR (95% CI)	1	0.89 (0.75–1.07)	0.83 (0.68–1.01)	**0.78 (0.64–0.96)**	**0.61 (0.48–0.78)**	**<0.01**	**0.88 (0.83–0.93)**
	Myocardial infarction	Deaths	147	113	109	110	84		
		HR (95% CI)	1	0.86 (0.66–1.12)	0.96 (0.73–1.26)	0.84 (0.63–1.12)	**0.67 (0.49–0.92)**	**0.02**	**0.89 (0.82–0.96)**
	Cerebrovascular disease	Deaths	274	197	180	158	134		
		HR (95% CI)	1	0.94 (0.77–1.14)	1.07 (0.87–1.32)	0.88 (0.70–1.10)	0.84 (0.65–1.07)	0.15	0.96 (0.90–1.02)
	Haemorrhagic stroke	Deaths	73	73	64	66	67		
		HR (95% CI)	1	0.98 (0.70–1.37)	0.99 (0.69–1.43)	0.88 (0.61–1.28)	0.89 (0.60–1.33)	0.48	0.97 (0.88–1.07)
	Ischemic stroke	Deaths	21	25	27	22	16		
		HR (95% CI)	1	0.99 (0.54–1.80)	1.10 (0.60–2.03)	0.77 (0.40–1.48)	0.59 (0.28–1.22)	0.10	0.86 (0.72–1.01)
Respiratory disease		Deaths	179	108	118	134	89		
		HR (95% CI)	1	0.72 (0.56–0.93)	0.95 (0.73–1.23)	0.98 (0.75–1.26)	0.75 (0.56–1.01)	0.31	0.96 (0.89–1.03)
Other cause of death		Deaths	568	470	442	436	461		
		HR (95% CI)	1	0.91 (0.80–1.04)	1.01 (0.88–1.16)	0.88 (0.76–1.01)	0.95 (0.82–1.11)	0.44	0.97 (0.94–1.01)

* Hazard ratios (HR) and 95% confidence intervals (95% CI) estimated in a Cox regression model stratified by centre, 1-year age and 5-year birth cohort categories, adjusted for education level (none,/primary school completed, technical/professional school, secondary school, university degree, not specified), smoking status (never smoker, former smoker who quit <10 years ago, former smoker who quit >10 years ago, former smoker, unknown when quit, current smoker of 1–14 cigarettes a day, current smoker of 15–24 cigarettes a day, current smoker of > = 25 cigarettes a day, current smoker but amount missing, smoking status unknown), physical activity (inactive, moderately inactive, moderately active, active), alcohol consumption (0, 0-<5, 5-<15, 15-<30, 30-<60, > = 60 g/day), weight (quintiles), intake of energy (kcals/day, continuous), menopausal status (pre, post-, peri-, unknown) and menopausal hormone use (yes, no, unknown).

† Median values of each category as continuous variable (cm).

Tables 4 and 5 shows the HRs for sitting height and all-cause and cause-specific mortality in men and women, respectively. We observed inverse associations for sitting height and all-cause mortality in both men and women (HR_Q5 vs. Q1_ = 0.81, 95%CI = 0.74–0.88, p for trend<0.01 in men; HR_Q5 vs. Q1_ = 0.86, 95%CI = 0.79–0.94, p for trend<0.01 in women). In contrast to the findings for overall height, we did not observe an association between sitting height and cancer mortality in either men or women. The associations between sitting height and circulatory disease mortality were similar to the inverse findings for overall height. In addition, we observed an inverse association between sitting height and death from haemorrhagic stroke in men only (HR_Q5 vs. Q1_ = 0.44, 95%CI = 0.23–0.84, p for trend<0.01) and from respiratory disease in men and women (HR_Q5 vs. Q1_ = 0.45, 95%CI = 0.28–0.71, p for trend <0.01; HR_Q5 vs. Q1_ = 0.60, 95%CI = 0.40–0.89, p for trend<0.01, respectively). When we analysed the association between sitting height and mortality, additional adjustment for overall height did not substantially change our results.

**Table 4:** Hazard ratios* and 95% confidence intervals for all cause and cause-specific mortality according to sitting height in men.

			Sitting height						
Cause of death			<87 cm	87-<89 cm	89-<91 cm	91-<93 cm	≧93cm	P for linear trend†	HR (95% CI) per 1 cm increase in height
MEN									
All-cause mortality		Person-years	190,524	155,166	193,151	192,945	271,451		
		Deaths	1,894	1,273	1,531	1,412	1,839		
		HR (95% CI)	1	**0.87 (0.81–0.94)**	**0.89 (0.83–0.96)**	**0.84 (0.78–0.91)**	**0.81 (0.74–0.88)**	**<0.01**	**0.98 (0.97–0.99)**
Cause-specific mortality		Person-years	183,369	148,567	184,265	183,540	255,934		
Cancer		Deaths	710	497	635	597	808		
		HR (95% CI)	1	0.91 (0.80–1.02)	1.01 (0.90–1.14)	1.01 (0.89–1.14)	1.07 (0.94–1.22)	0.29	1.01 (0.995–1.02)
	Smoking-related cancer	Deaths	303	229	294	236	352		
		HR (95% CI)	1	1.28 (0.97–1.69)	1.21 (0.93–1.58)	1.32 (0.99–1.76)	1.30 (0.98–1.73)	0.66	1.01 (0.98–1.03)
	Non smoking-related cancer	Deaths	407	268	341	361	456		
		HR (95% CI)	1	0.82 (0.66–1.02)	0.83 (0.67–1.02)	0.90 (0.73–1.12)	**0.78 (0.63–0.97)**	0.09	0.98 (0.96–1.00)
Circulatory disease		Deaths	615	367	425	353	443		
		HR (95% CI)	1	**0.81 (0.70–0.93)**	**0.81 (0.70–0.93)**	**0.69 (0.60–0.81)**	**0.64 (0.55–0.75)**	**<0.01**	**0.96 (0.94–0.97)**
	Ischaemic heart disease	Deaths	291	192	219	176	221		
		HR (95% CI)	1	0.84 (0.69–1.02)	**0.81 (0.67–0.99)**	**0.67 (0.54–0.83)**	**0.62 (0.50–0.78)**	**<0.01**	**0.95 (0.93–0.97)**
	Myocardial infarction	Deaths	159	101	119	100	113		
		HR (95% CI)	1	0.86 (0.66–1.12)	0.88 (0.67–1.15)	0.77 (0.57–1.03)	**0.63 (0.47–0.86)**	**<0.01**	**0.95 (0.93–0.98)**
	Cerebrovascular disease	Deaths	139	61	80	53	63		
		HR (95% CI)	1	**0.72 (0.52–0.98)**	0.87 (0.64–1.19)	**0.62 (0.43–0.89)**	**0.56 (0.38–0.82)**	**<0.01**	**0.96 (0.93–0.99)**
	Haemorrhagic stroke	Deaths	36	22	29	20	26		
		HR (95% CI)	1	0.75 (0.43–1.32)	0.80 (0.46–1.39)	**0.53 (0.28–0.99)**	**0.44 (0.23–0.84)**	**<0.01**	**0.93 (0.88–0.98)**
	Ischemic stroke	Deaths	18	3	10	6	12		
		HR (95% CI)	1	0.24 (0.07–0.85)	0.71 (0.29–1.72)	0.46 (0.16–1.35)	0.72 (0.26–1.97)	0.85	0.99 (0.91–1.09)
Respiratory disease		Deaths	110	60	41	46	39		
		HR (95% CI)	1	0.85 (0.60–1.19)	**0.52 (0.35–0.78)**	0.67 (0.45–1.01)	**0.45 (0.28–0.71)**	**<0.01**	**0.93 (0.90–0.96)**
Other cause of death		Deaths	375	265	308	297	345		
		HR (95% CI)	1	0.87 (0.74–1.03)	**0.82 (0.69–0.97)**	**0.79 (0.66–0.94)**	**0.64 (0.53–0.77)**	**<0.01**	**0.96 (0.94–0.97)**

* Hazard ratios (HR) and 95% confidence intervals (95% CI) estimated in a Cox regression model stratified by centre, 1-year age and 5-year birth cohort categories, adjusted for education level (none,/primary school completed, technical/professional school, secondary school, university degree, not specified), smoking status (never smoker, former smoker who quit <10 years ago, former smoker who quit >10 years ago, former smoker, unknown when quit, current smoker of 1–14 cigarettes a day, current smoker of 15–24 cigarettes a day, current smoker of > = 25 cigarettes a day, current smoker but amount missing, smoking status unknown), physical activity (inactive, moderately inactive, moderately active, active), alcohol consumption (0, 0-<5, 5-<15, 15-<30, 30-<60, > = 60 g/day), weight (quintiles), intake of energy (kcals/day, continuous).

† Median values of each category as continuous variable (cm).

**Table 5:** Hazard ratios* and 95% confidence intervals for all-cause and cause-specific mortality according to sitting height in women.

			Sitting height						
Cause of death			<82 cm	82-<84 cm	84-<86 cm	86-<88 cm	≧88cm	P for linear trend†	HR (95% CI) per 1 cm increase in height
All-cause mortality		Person-years	336,999	329,496	426,977	404,731	484,850		
		Deaths	1,582	1,332	1,674	1,467	1,626		
		HR (95% CI)	1	0.95 (0.88–1.03)	0.94 (0.87–1.02)	**0.90 (0.83–0.97)**	**0.86 (0.79–0.94)**	**<0.01**	**0.99 (0.98–0.99)**
									
Cause-specific mortality		Person-years	320,489	311,699	402,879	381,913	459,258		
Cancer		Deaths	614	606	822	730	890		
		HR (95% CI)	1	1.08 (0.96–1.21)	1.13 (1.01–1.27)	1.07 (0.94–1.20)	1.08 (0.95–1.22)	0.26	1.01 (0.995–1.02)
	Smoking-related cancer	Deaths	292	242	352	292	344		
		HR (95% CI)	1	0.78 (0.59–1.03)	0.78 (0.59–1.03)	0.86 (0.64–1.14)	0.71 (0.53–0.95)	0.12	0.98 (0.96–1.01)
	Non-smoking-related cancer	Deaths	322	364	470	438	546		
		HR (95% CI)	1	1.09 (0.87–1.37)	1.04 (0.83–1.30)	0.96 (0.77–1.21)	1.15 (0.91–1.44)	0.15	1.01 (0.995–1.03)
Circulatory disease		Deaths	442	264	294	230	223		
		HR (95% CI)	1	**0.80 (0.68–0.94)**	**0.77 (0.65–0.91)**	**0.69 (0.57–0.83)**	**0.60 (0.49–0.74)**	**<0.01**	**0.95 (0.94–0.97)**
	Ischaemic heart disease	Deaths	136	79	86	63	57		
		HR (95% CI)	1	0.77 (0.57–1.03)	**0.69 (0.51–0.94)**	**0.59 (0.42–0.83)**	**0.47 (0.33–0.69)**	**<0.01**	**0.94 (0.91–0.97)**
	Myocardial infarction	Deaths	69	46	61	44	40		
		HR (95% CI)	1	0.85 (0.57–1.26)	0.87 (0.59–1.29)	0.71 (0.46–1.10)	**0.54 (0.34–0.87)**	**<0.01**	**0.94 (0.90–0.98)**
	Cerebrovascular disease	Deaths	131	90	92	68	67		
		HR (95% CI)	1	0.96 (0.72–1.28)	0.86 (0.64–1.16)	0.73 (0.52–1.02)	**0.64 (0.44–0.92)**	**0.02**	**0.96 (0.94–0.99)**
	Haemorrhagic stroke	Deaths	38	36	35	34	43		
		HR (95% CI)	1	0.93 (0.57–1.50)	0.73 (0.44–1.20)	0.75 (0.45–1.27)	0.79 (0.46–1.35)	0.28	0.97 (0.93–1.02)
	Ischemic stroke	Deaths	10	10	13	9	6		
		HR (95% CI)	1	0.91 (0.36–2.28)	0.86 (0.35–2.12)	0.64 (0.24–1.75)	0.40 (0.13–1.24)	0.10	0.92 (0.84–1.01)
Respiratory disease		Deaths	89	47	69	55	61		
		HR (95% CI)	1	**0.58 (0.40–0.84)**	**0.66 (0.46–0.93)**	**0.59 (0.40–0.86)**	**0.60 (0.40–0.89)**	**<0.01**	**0.95 (0.92–0.98)**
Other cause of death		Deaths	296	281	298	275	287		
		HR (95% CI)	1	1.06 (0.89–1.26)	0.88 (0.74–1.06)	0.90 (0.75–1.09)	**0.81 (0.66–0.98)**	**<0.01**	**0.98 (0.96–0.995)**

* Hazard ratios (HR) and 95% confidence intervals (95% CI) estimated in a Cox regression model stratified by centre, 1-year age and 5-year birth cohort categories, adjusted for education level (none,/primary school completed, technical/professional school, secondary school, university degree, not specified), smoking status (never smoker, former smoker who quit <10 years ago, former smoker who quit >10 years ago, former smoker, unknown when quit, current smoker of 1–14 cigarettes a day, current smoker of 15–24 cigarettes a day, current smoker of > = 25 cigarettes a day, current smoker but amount missing, smoking status unknown), physical activity (inactive, moderately inactive, moderately active, active), alcohol consumption (0, 0-<5, 5-<15, 15-<30, 30-<60, > = 60 g/day), weight (quintiles), energy intake (kcals/day, continuous), menopausal status (pre, post-, peri-, unknown) and menopausal hormone use (yes, no, unknown).

† Median values of each category as continuous variable (cm).

We also investigated interactions between sitting height and overall height in relation to mortality (Table 6). Taller overall height and taller sitting height was strongly inversely associated with death from respiratory disease in men (P_interaction_ = 0.03) but not women. However, there were no significant interactions between sitting height and overall height in relation to all-cause, cancer or circulatory disease mortality. Interaction terms for overall height and mortality by age, smoking status (smoker/non-smoker), and alcohol intake (high/low) did not reveal meaningful differences; however, for BMI, we observed an increased risk per 5cm increment in height for all-cause mortality only among women with a BMI <25 kg/m^2^ (data not shown).

**Table 6:** Hazard ratios* and 95% confidence intervals for all-cause and cause-specific mortality according to sitting height and overall height in men and women.

		Overall height (tertiles)			
		Lowest (<170 cm)	Middle (170-<176 cm)	Highest (≧92 cm)	P_interaction_
MEN					
All-cause mortality	Lowest (<89 cm)	1	0.89 (0.82–0.97)	1.04 (0.87–1.24)	
	Middle (89-<92 cm)	0.99 (0.90–1.08)	0.88 (0.82–0.96)	0.81 (0.73–0.90)	0.58
	Highest (≧92 cm)	0.99 (0.79–1.25)	0.91 (0.83–1.00)	0.83 (0.76–0.90)	
All cancer	Lowest (<89 cm)	1	0.93 (0.81–1.07)	1.18 (0.89–1.56)	
	Middle (89-<92 cm)	1.08 (0.93–1.26)	1.03 (0.91–1.16)	0.98 (0.83–1.15)	0.77
	Highest (≧92 cm)	1.03 (0.71–1.48)	1.18 (1.02–1.36)	1.08 (0.95–1.23)	
Circulatory disease	Lowest (<89 cm)	1	0.75 (0.64–0.89)	0.74 (0.50–1.08)	
	Middle (89-<92 cm)	0.95 (0.80–1.12)	0.75 (0.65–0.87)	0.58 (0.47–0.72)	0.53
	Highest (≧92 cm)	0.91 (0.60–1.39)	0.70 (0.58–0.84)	0.62 (0.53–0.73)	
Respiratory disease	Lowest (<89 cm)	1	1.27 (0.87–1.84)	1.22 (0.48–3.09)	
	Middle (89-<92 cm)	0.79 (0.49–1.28)	0.69 (0.45–1.04)	0.36 (0.17–0.75)	**0.03**
	Highest (≧92 cm)	0.71 (0.17–2.92)	0.84 (0.51–1.38)	0.50 (0.32–0.80)	
	Sitting height (tertiles)	Lowest (<158cm)	Middle (158-<163 cm)	Highest (≧163 cm)	P_interaction_
WOMEN					
All-cause mortality	Lowest (<84 cm)	1	1.07 (0.98–1.16)	1.15 (0.95–1.39)	
	Middle (84-<87 cm)	0.91 (0.82–1.00)	1.00 (0.93–1.08)	1.02 (0.93–1.12)	0.29
	Highest (≧87 cm)	1.04 (0.83–1.30)	0.91 (0.82–1.01)	0.93 (0.86–1.01)	
All cancer	Lowest (<84 cm)	1	1.07 (0.94–1.23)	1.19 (0.91–1.55)	
	Middle (84-<87 cm)	1.04 (0.89–1.20)	1.14 (1.02–1.27)	1.08 (0.94–1.25)	0.66
	Highest (≧87 cm)	1.24 (0.91–1.68)	0.99 (0.85–1.15)	1.09 (0.97–1.23)	
Circulatory disease	Lowest (<84 cm)	1	1.19 (0.99–1.44)	1.30 (0.85–1.98)	
	Middle (84-<87 cm)	0.84 (0.67–1.06)	0.89 (0.74–1.07)	0.88 (0.70–1.12)	0.05
	Highest (≧87 cm)	0.97 (0.59–1.60)	0.86 (0.67–1.09)	0.75 (0.62–0.91)	
Respiratory disease	Lowest (<84 cm)	1	0.67 (0.43–1.10)	1.43 (0.64–3.16)	
	Middle (84-<87 cm)	0.46 (0.25–0.84)	0.85 (0.59–1.22)	1.07 (0.70–1.62)	0.11
	Highest (≧87 cm)	0.31 (0.04–2.25)	0.72 (0.43–1.19)	0.71 (0.48–1.05)	

* Hazard ratios (HR) and 95% confidence intervals (95% CI) estimated in a Cox regression model stratified by centre, 1-year age and 5-year birth cohort categories, adjusted for education level (none,/primary school completed, technical/professional school, secondary school, university degree, not specified), smoking status (never smoker, former smoker who quit <10 years ago, former smoker who quit >10 years ago, former smoker, unknown when quit, current smoker of 1–14 cigarettes a day, current smoker of 15–24 cigarettes a day, current smoker of > = 25 cigarettes a day, current smoker but amount missing, smoking status unknown), physical activity (inactive, moderately inactive, moderately active, active), alcohol consumption (0, 0-<5, 5-<15, 15-<30, 30-<60, > = 60 g/day), weight (quintiles), energy intake (kcals/day, continuous). Models in women were further adjusted by menopausal status (pre, post-, peri-, unknown) and menopausal hormone use (yes, no, unknown).

After Bonferroni correction, the significance of our results was not substantially changed.

## Discussion

In this large prospective study, overall height was positively associated with deaths from cancer, but inversely associated with deaths from circulatory disease. These results are supported by a previous meta-analysis of 1 million people from 121 prospective studies [6]. In the present study, sitting height was not associated with cancer mortality but was inversely associated with all-cause mortality, circulatory deaths, and death from respiratory disease. To our knowledge, this is the first study to report an inverse association between sitting height and death from respiratory disease. The World Cancer Research Fund/ American Institute for Cancer Research (WCRF/AICR) reported that there is convincing data that height increases the risk of individuals being diagnosed with cancers of the colorectum, breast (postmenopausal) and ovary [20, 21]; furthermore, height ‘*probably’* increases the risk of cancers of the pancreas and breast (premenopausal). To complement this previous data on incidence, our data suggests a role for height and risk of cancer mortality.

Short stature is a well-documented risk factor for mortality from circulatory diseases [6, 9, 22], ischemic heart disease [23], ischemic stroke [22, 24, 25] and haemorrhagic stroke [24, 25] in previous studies. The results of this current analysis corroborate these prior studies but we also observed strong inverse associations between height and subtypes of circulatory disease death despite their different pathologies.

Whether a relationship between sitting height and mortality also exists is largely unknown. Wang et al. reported height and sitting height were positively associated with cancer death, but were inversely associated with death from cardiovascular disease in a cohort of 135,000 Chinese men and women [9]. Four other studies reported no association between sitting height and mortality [7, 10–12]. Our study in a large European population generally supports the reports from the Chinese, although we did not find a positive association between sitting height specifically and cancer mortality.

There are several potential underlying mechanisms to explain the opposing association of adult height with circulatory disease and cancer mortality. The positive association between adult height and mortality from cancer may be a result of taller people having larger organs, and a greater number of cells at risk of malignant transformation and/or proliferation [26]. Furthermore, attained adult height is known to be related to early nutrition in childhood or adolescence [3, 5]. In contrast, the inverse association between height and cardiovascular disease mortality has been proposed to be due to taller people and people with higher sitting height having larger coronary vessel diameters and a slower heart rate and/or greater lung capacity [8, 27–30]. Height may also be a marker of early exposure to components of the insulin/growth hormone axis. Height is correlated with circulating levels of insulin-like growth factor (IGF)-I, the main mediator of growth hormone activity and a hormone that has been positively associated with cancers at a number of anatomic sites [31–35], but IGF-1 levels are generally inversely related with circulatory disease risk [36–40]. Crowe et al. reported that each 10cm increase in height corresponded to a 4% increase in circulating IGF-1 levels [41]; therefore, increasing IGF-1 levels might mediate the opposing effect of height on cancer and circulatory disease mortality. To clarify the underlying mechanisms, further studies are needed to investigate IGF-1 levels in relation to cause-specific mortality risk while accounting for adult height. Furthermore, several genetic factors are related with height, cancer and cardiovascular disease [42, 43]. Identifying such genetic variants might shed light on potential mechanisms underlying the associations between height and mortality.

Davey Smith et al. reported that sitting height was strongly positively associated with insulin resistance [7]; thus, we expected a clearer association between sitting height and cancer mortality than overall height. Despite finding a positive association between overall height and cancer mortality in our data, there was no association for sitting height. These null findings for sitting height and cancer mortality may be plausible because despite the association with insulin resistance, sitting height has been associated with improved prognosis in cancer survivors due to better lung function in those with greater sitting height [8, 44]. Our finding that sitting height was inversely associated with death from respiratory disease is of note as this may be due to the aforementioned association between sitting height and lung function [8, 44].

Height is positively associated with education level among women in this study. Previous studies reported that lower educational levels have been associated with increased mortality, and incidence of coronary heart disease and stroke in Europe and the United States [45–47]. In an attempt to control for this potential confounder, we adjusted our models for educational level, although our results did not change from the unadjusted models.

A major strength of the EPIC study is the large study population representing findings from multiple countries and its long follow-up, resulting in a large number of deaths allowing us to analyse and distinguish between different causes of death. This study enabled us to examine measured height on the majority of participants, to adjust the self-reported height variable in the others, and to examine measured sitting height in a large subset of the cohort. In contrast, this study had some limitations. With a large body of information on lifestyle variables, we could adjust for many potential confounding factors, although the possibility of residual confounding cannot be excluded. Additionally, we divided cardiovascular disease into subgroups, which may result in some degree of misclassification.

In conclusion, this study revealed opposing findings for the relationship between height on cancer and circulatory disease mortality. Specifically, we showed that height was positively associated with death from cancer, but inversely associated with death from circulatory disease. Furthermore, this is the first study to show the inverse association between sitting height and death from respiratory disease. These findings could be used to contribute to risk prediction models to target individuals for specific screening programmes.

## Abbreviations

BMI: body mass index
CI: confidence interval
EPIC: European Prospective Investigation into Cancer and Nutrition
HR: hazard ratio
IARC: International Agency for Research on Cancer
ICD: International Classification of Diseases
IGF: insulin-like growth factor
WCRF/AICR: World Cancer Research Fund/American Institute of Cancer Research
